# Structural basis for activation of Arf1 at the Golgi complex

**DOI:** 10.1016/j.celrep.2022.111282

**Published:** 2022-08-30

**Authors:** Arnold J. Muccini, Margaret A. Gustafson, J. Christopher Fromme

**Affiliations:** 1 Department of Molecular Biology and Genetics, Weill Institute for Cell and Molecular Biology, Cornell University, Ithaca, NY 14853, USA; 2 Present address: Mitochondrial DNA Replication Group, Genome Integrity and Structural Biology Laboratory, National Institute of Environmental Health Sciences (NIEHS), NIH, Research Triangle Park, NC 27709, USA; 3 Lead contact

## Abstract

The Golgi complex is the central sorting station of the eukaryotic secretory pathway. Traffic through the Golgi requires activation of Arf guanosine triphosphatases that orchestrate cargo sorting and vesicle formation by recruiting an array of effector proteins. Arf activation and Golgi membrane association is controlled by large guanine nucleotide exchange factors (GEFs) possessing multiple conserved regulatory domains. Here we present cryoelectron microscopy (cryoEM) structures of full-length Gea2, the yeast paralog of the human Arf-GEF GBF1, that reveal the organization of these regulatory domains and explain how Gea2 binds to the Golgi membrane surface. We find that the GEF domain adopts two different conformations compatible with different stages of the Arf activation reaction. The structure of a Gea2-Arf1 activation intermediate suggests that the movement of the GEF domain primes Arf1 for membrane insertion upon guanosine triphosphate binding. We propose that conformational switching of Gea2 during the nucleotide exchange reaction promotes membrane insertion of Arf1.

## In brief

Arf1 is a GTPase that regulates Golgi trafficking by recruiting many effector proteins. Muccini et al. report cryoEM structures of the Arf1 activator Gea2, capturing Gea2 in multiple conformational states including a Gea2-Arf1 activation intermediate. The structures help explain how Gea2 activates Arf1 on the Golgi membrane surface.

## Graphical Abstract

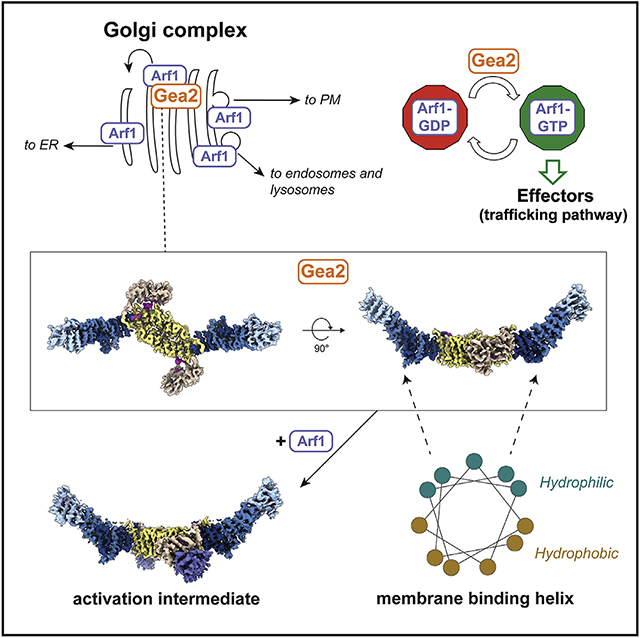

## INTRODUCTION

The endomembrane system provides essential compartmentalization for all eukaryotic cells. Most transmembrane and lumenal proteins are synthesized at the ER and then travel through the secretory pathway to reach their target organelle. At the center of the secretory pathway is the Golgi complex, which modifies secretory proteins and serves as a trafficking hub. Arf1 and its close paralogs are essential regulators of cargo sorting and vesicle formation at the Golgi complex that function by recruiting a large number of prominent effectors including coat protein complex I (COPI)/coatomer, clathrin cargo adaptors, lipid signaling enzymes, vesicle tethers, and regulators of other pathways (Adarska et al., 2021; Cherfils, 2014; Donaldson and Jackson, 2011; Gillingham and Munro, 2007). Arf1 is a guanosine triphosphatase (GTPase), cycling between an inactive guanosine diphosphate (GDP)-bound state and an active guanosine triphosphate (GTP)-bound state (Kahn and Gilman, 1986). Arf1 possesses an N-terminal myristoylated amphipathic helix that anchors it to the Golgi membrane (Haun et al., 1993; Kahn et al., 1988). When GDP-bound, this membrane-binding feature is masked and Arf1 is cytosolic. When Arf1 is activated to its GTP-bound state, a change in conformation exposes the myristoylated amphipathic helix, resulting in stable membrane association (Amor et al., 1994; Antonny et al., 1997; Franco et al., 1995; Goldberg, 1998). The active conformation of Arf1 is therefore required to recruit its numerous effectors to the Golgi membrane surface.

Arf1 activation in cells requires nucleotide exchange by specific guanine nucleotide exchange factors (GEFs). Arf1 is activated at the Golgi complex by at least two distinct but related Arf-GEFs, GBF1 and BIG1/2 (Claude et al., 1999; Togawa et al., 1999). The budding yeast homolog of BIG1/2 is Sec7, which localizes to late Golgi compartments and activates Arf1 to control trafficking to endosomes, lysosomes, earlier Golgi compartments, and the plasma membrane (Franzusoff et al., 1991; Novick et al., 1981). The budding yeast homologs of GBF1, named Gea1 and Gea2, localize to early and medial Golgi compartments where Arf1 activation orchestrates the formation of COPI vesicles destined for the ER and earlier Golgi compartments (Gustafson and Fromme, 2017; Peyroche et al., 1996; Spang et al., 2001).

The Golgi Arf-GEFs share a homologous catalytic GEF domain, referred to as a “Sec7” domain, with members of other Arf-GEF families (Casanova, 2007). The structural and biochemical basis for nucleotide exchange by Sec7 GEF domains is well established and involves remodeling of the Arf1 nucleotide-binding site by interaction with the GEF (Goldberg, 1998; Renault et al., 2003). The ARNO/Cytohesin/Grp1 and BRAG/IQSec7 Arf-GEFs possess structurally characterized pleckstrin homology (PH) domains that direct membrane binding and regulation of GEF activity (Aizel et al., 2013; Cronin et al., 2004; Das et al., 2019; DiNitto et al., 2007; Malaby et al., 2018). In contrast, the Golgi-localized “large” Arf-GEFs do not contain PH domains and instead contain multiple regulatory domains that are conserved across species but are not found in other proteins (Bui et al., 2009; Mouratou et al., 2005). Previous studies have dissected the biochemical and cell biological roles of these regulatory domains and have identified which domains are required for Golgi membrane binding and activation of Arf1 (Bouvet et al., 2013; Christis and Munro, 2012; Gustafson and Fromme, 2017; Meissner et al., 2018; Pocognoni et al., 2018; Richardson and Fromme, 2012; Richardson et al., 2012). Structures are available for the N-terminal “DCB-HUS” domains in isolation (Galindo et al., 2016; Richardson et al., 2016; Wang et al., 2016), but the lack of structural information for the full-length proteins has prevented an understanding of how the regulatory domains function together with the GEF domain during Arf1 activation.

Here, we present cryoelectron microscopy (cryoEM) structures of full-length Gea2 and a Gea2-Arf1 activation intermediate. These structures reveal the organization of the regulatory domains within the Gea2 dimer. We identify two conserved structural elements in Gea2: an amphipathic helix between the HDS1 and HDS2 domains that is required for membrane binding and an ordered linker between the GEF and HDS1 domains. Unexpectedly, the GEF domain of Gea2 adopts two conformational states. Structural analysis indicates that the GEF-HDS1 linker plays a role in conformational switching: the “closed” state of the GEF domain is compatible with initial binding to Arf1-GDP but incompatible with subsequent binding to nucleotide-free Arf1 because of a steric clash between nucleotide-free Arf1 and the linker. The structural data therefore suggest that the Arf1 nucleotide exchange reaction involves conformational change of its GEF from the closed state to the “open” state. Based on the orientation of Gea2 on the membrane, this GEF conformational change appears to directly couple Arf1 activation to membrane insertion.

## RESULTS

### Architecture of the Gea2 homodimer

**Figure 1.**
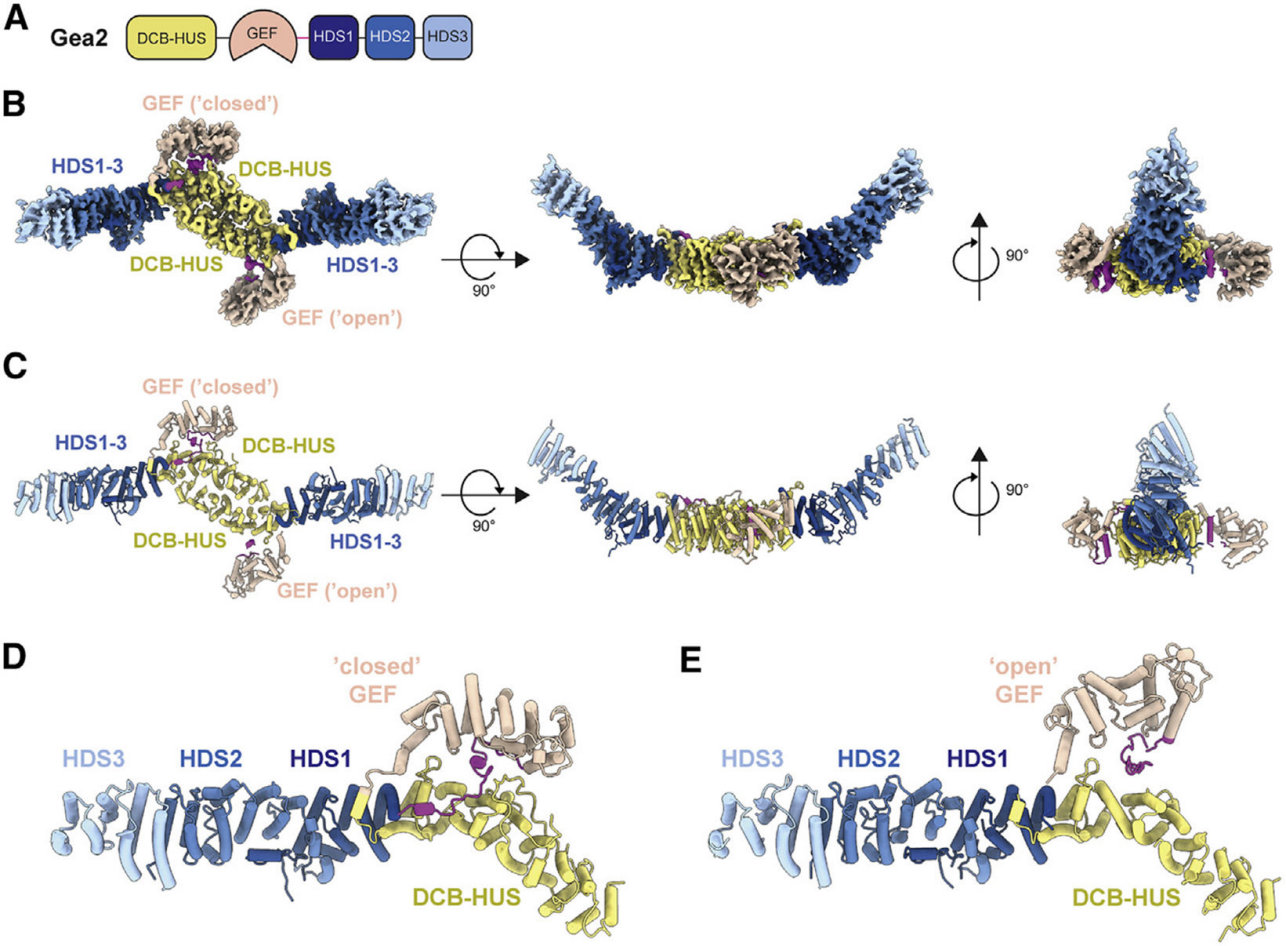
Structure of Gea2 determined by cryoEM (A) Schematic of Gea2 primary structure indicating conserved domains. DCB, dimerization and cyclophilin binding; HUS, homology upstream of Sec7; GEF, guanine nucleotide exchange factor (also known as “Sec7 domain”); HDS, homology downstream of Sec7. (B) CryoEM density of the Gea2 dimer in its closed/open conformation. One monomer adopts an open conformation of the GEF domain and the other monomer adopts a closed conformation. The GEF-HDS1 linker is colored magenta. (C) Atomic model of the Gea2 dimer, shown in cartoon depiction. (D) Close-up view of the closed monomer. (E) Close-up view of the open monomer. See also Figures S1-S5 and S7.

Gea2 and its paralogs possess an N-terminal DCB-HUS regulatory domain and C-terminal HDS1, HDS2, and HDS3 regulatory domains (Mouratou et al., 2005; Richardson et al., 2016) (Figure 1A). We produced full-length *Saccharomyces cerevisiae* Gea2 by overexpression in *Pichia pastoris* (Figure S1A) and determined its structure using cryoEM (Figures S2 and S3). Three-dimensional classification of the particles revealed three distinct conformations of Gea2 homodimers that differed only in the positioning of the GEF domain, with each monomer adopting either a “closed” or “open” position relative to the regulatory domains (Figure S2). Based on the relative numbers of particle images that sorted into each of these three classes (~30% “closed/closed,” ~30% “open/open,” and ~40% “closed/open”), the conformation adopted by each monomer within the dimer appears to be largely independent of that of its binding partner. We took advantage of the 2-fold symmetry of the Gea2 homodimer by using symmetry expansion and focused refinements during data processing (see Figures S2 and S3; STAR Methods) to obtain higher-resolution maps for the closed and open monomers and for the three different dimeric states (Figures 1B and S4). These maps were then used to build and refine atomic models (Figures 1C-1E and S4; Table 1). We begin our description of the structure using the “closed/open” dimer, as it exhibits both the closed (Figure 1D) and open (Figure 1E) states of the GEF domain.

The HDS1, −2, and −3 domains form an extended helical repeat structure that is contiguous with the DCB-HUS domain, such that the HDS3 domains of each monomer lie at the distal ends of the homodimer (Figures 1B and 1C). The GEF domain lies adjacent to the HUS domain and is connected to the HUS and HDS1 domains through ordered linker regions (Figure S5). The “HUS box,” which is a conserved region near the C-terminal end of the HUS domain (Mouratou et al., 2005), interacts directly with the HUS-GEF linker, which is simply an extension of the first α helix of the GEF domain (Figures S5E-S5G). Temperature-sensitive mutations have been identified in the region surrounding the HUS box (Park et al., 2005), lending support to the importance of this interaction. The linker that connects the GEF domain to the HDS1 domain (GEF-HDS1 linker) comprises ~45 conserved ordered residues and is discussed in further detail below (Figures S5A-S5C).

**Figure 2.**
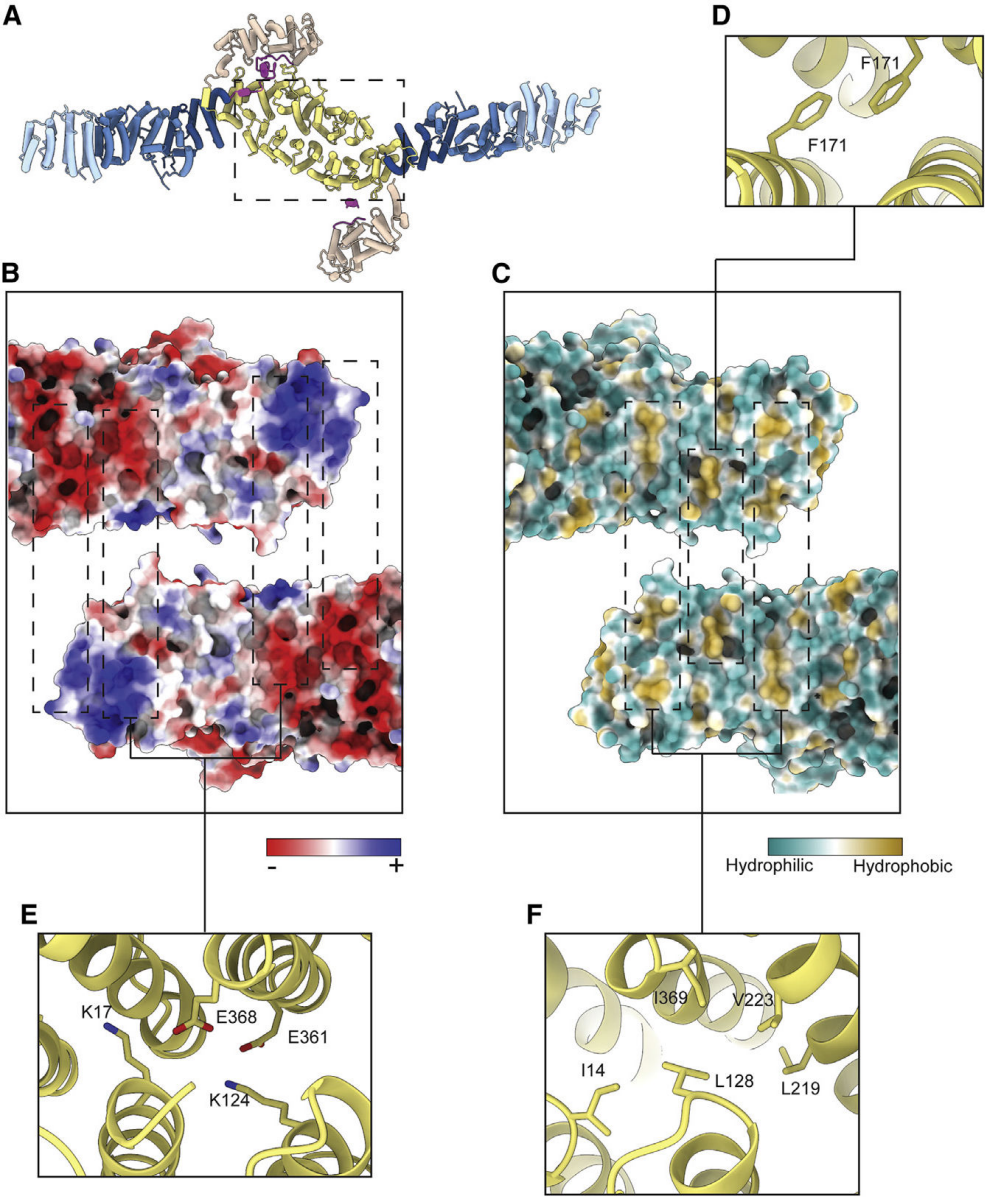
Gea2 dimerizes via the DCB-HUS domains (A) Gea2 dimer, with a dashed box indicating the region depicted in (B) and (C). (B) View of the dimerization interface peeled apart and colored by calculated charge potential. (C) View of the dimerization interface peeled apart and colored by hydrophobicity. (D) Close-up view highlighting a homotypic hydrophobic interaction at the dimer interface. (E) Close-up view highlighting electrostatic interactions at the dimer interface. (F) Close-up view highlighting hydrophobic interactions at the dimer interface.

Dimerization occurs through extensive hydrophobic, polar, and electrostatic interactions between the DCB-HUS domains of each monomer (Figures 2A-2F), consistent with the established role of this domain for dimerization of Gea2/GBF1 homologs (Bhatt et al., 2016; Grebe et al., 2000; Ramaen et al., 2007). The fold of the Gea2 DCB-HUS domain is quite similar to that of the distinct Arf-GEF Sec7 (Richardson et al., 2016), although this domain does not appear to mediate dimerization of Sec7. Previous studies identified substitution mutations in the DCB subdomain of GBF1 that disrupted its dimerization in residues corresponding to K124 and D163 in Gea2 (Bhatt et al., 2016; Ramaen et al., 2007). Examination of the dimerization interface indicates that K124 is involved in favorable interactions between monomers (Figure 2E). Therefore, the observed dimerization interface is supported by these published functional results and is likely conserved across Gea2/GBF1 paralogs in different species.

### Gea2 binds to the Golgi via a conserved amphipathic helix

Several Arf-GEFs possess PH domains that bind to membranes via specific interactions with phosphoinositide lipids (Casanova, 2007). The Golgi Arf-GEFs do not possess a PH domain, and although the HDS1, −2, and −3 domains are known to be important for Golgi localization of Gea1/Gea2 and GBF1 (Bouvet et al., 2013; Gustafson and Fromme, 2017; Meissner et al., 2018; Pocognoni et al., 2018), their membrane-binding mechanism is unknown.

**Figure 3.**
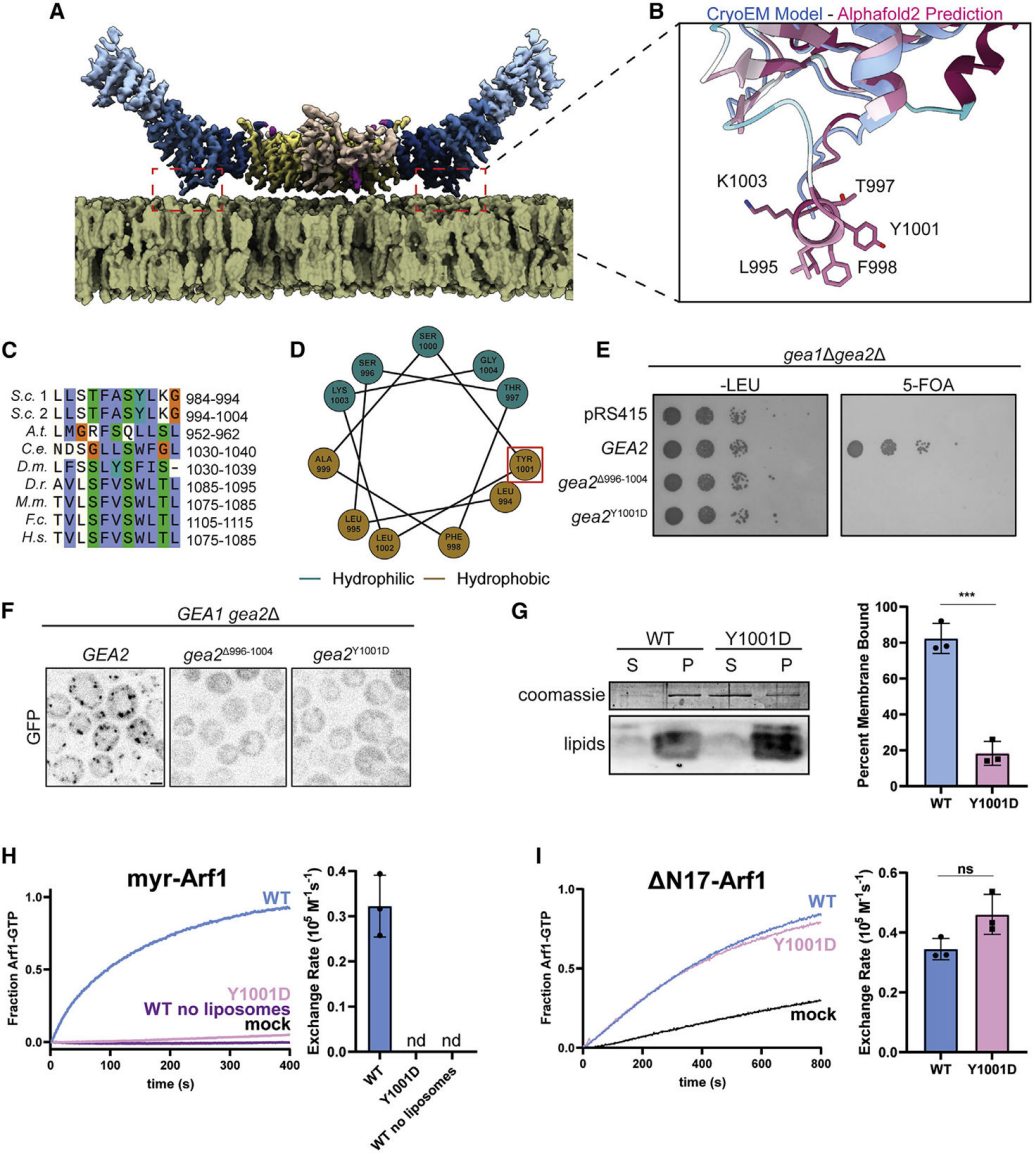
A conserved amphipathic α helix mediates Gea2 membrane binding (A) Gea2 depicted on a modeled membrane surface. (B) Close-up view of the amphipathic helix predicted by both secondary and tertiary structure prediction methods but absent from the experimentally determined cryoEM density. The structural model determined by cryoEM is superimposed onto the AlphaFold prediction (Jumper et al., 2021). The AlphaFold prediction is colored by conservation, with dark red representing the most conserved residues and cyan representing the least conserved residues. (C) Sequence alignment highlighting conservation of the helix; colors highlight conserved residues based on their biochemical properties. (D) Helical wheel indicating the amphipathic nature of the helix. Red box indicates Tyr residue mutated for functional experiments. (E) *GEA2* complementation test (plasmid shuffling). (F) Localization analysis of Gea2 and amphipathic helix mutants. Scale bar, 2 μm. (G) *In vitro* membrane-binding assay (liposome pelleting) using purified proteins and synthetic liposomes. S, supernatant; P, pellet. ***p < 0.001. (H) *In vitro* GEF activity assay using purified Gea2 proteins (200 nM), purified myristoylated-Arf1 substrate (1 μM), and synthetic liposomes. nd, not detectable. (I) *In vitro* GEF activity assay using purified Gea2 proteins (25 nM) and the ΔN17-Arf1 substrate (500 nM) without liposomes. ns, not significant. For data quantitation in (G), (H), and (I), data are presented as mean (bars) and individual data values (closed circles). Error bars are 95% confidence intervals.

Analysis of the Gea2 cryoEM structures revealed the presence of an unstructured but conserved sequence in the linker between the HDS1 and HDS2 domains (Figures 3A-3C). This sequence is predicted to form an amphipathic helix by both secondary and tertiary sequence prediction methods (Figure 3D). We reasoned that its conservation, position, and flexible connection to the rest of the protein made this sequence a strong candidate for a membrane-inserting amphipathic helix (Drin and Antonny, 2010). We note that this helix is distinct from amphipathic helices in the HDS1 and HDS2 domains previously proposed by other groups to be important for membrane binding. Our structural data indicate that the amphipathic helices previously studied by others are instead part of the core helical repeat structure of these domains. As the hydrophobic faces of these helices are buried within the hydrophobic protein interior, they are unavailable for membrane interaction.

To test the role and importance of this amphipathic α helix, we produced two different mutants of Gea2, one in which this helix was removed, Δ996-1004, and another in which a Tyr residue was substituted with Asp, Y1001D. This Tyr residue lies at a position in the primary sequence which has conserved hydrophobic character across evolution (Figure 3C). We found that both the Δ996-1004 and Y1001D mutants lost their ability to support cell growth, despite being expressed at endogenous levels (Figures 3E and S1B). We also observed that these mutant proteins lost their localization to the Golgi complex, localizing instead to the cytoplasm (Figure 3F). These results indicate that this conserved amphipathic helix is required for Golgi membrane association *in vivo.*

To determine whether this amphipathic helix is involved in direct interaction between Gea2 and the membrane surface, we purified the Gea2 Y1001D mutant protein (Figure S1C) and tested its ability to interact with liposome membranes *in vitro.* Using a lipid mix that wild-type Gea2 associates with robustly, we found that the Y1001D mutant protein exhibited a dramatic reduction in membrane-binding capability *in vitro* (Figure 3G). This indicates that the amphipathic helix is directly involved in Gea2 membrane binding.

To determine whether the amphipathic helix is required for membrane-proximal Arf1 activation, we employed an established *in vitro* GEF assay for Gea2 (Gustafson and Fromme, 2017). We found that purified Gea2 Y1001D was well behaved biochemically but unable to activate full-length myristoylated-Arf1 on liposome membranes (Figures 3H and S1D). A similar lack of activation was seen when liposomes were omitted from reactions with wild-type Gea2 (Figure 3H). Importantly, Gea2 Y1001D retained robust GEF activity toward ΔN17-Arf1 in the absence of liposome membranes (Figures 3I and S1E). ΔN17-Arf1 is a truncated form of Arf1 that lacks its N-terminal amphipathic helix and therefore does not need to insert into membranes in order to be activated (Kahn et al., 1992; Paris et al., 1997). These results indicate that the Gea2 amphipathic helix is specifically required for activating Arf1 on the membrane surface.

Taken together, our results indicate that Gea2 uses the conserved amphipathic helix in the HDS1-HDS2 linker to bind to the Golgi membrane surface in order to activate Arf1. The dimeric nature of Gea2 enables us to model its orientation on the membrane with high confidence (Figure 3A). These findings also highlight how Arf1 activation and insertion of its myristoylated N-terminal helix into a membrane are intimately coupled.

### Gea2 adopts an open conformation when bound to nucleotide-free Arf1

**Figure 4.**
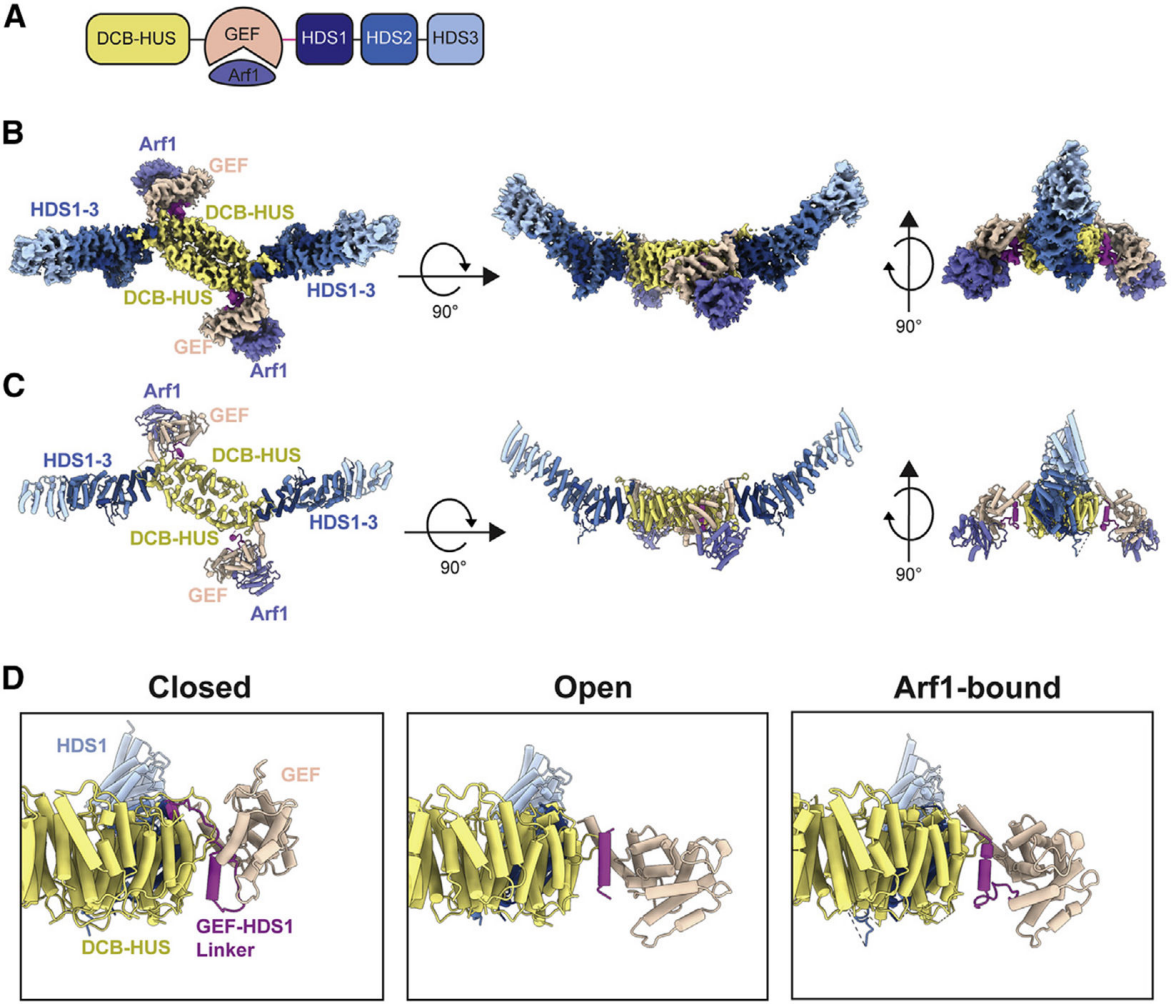
CryoEM structure of a Gea2-Arf1 activation intermediate complex (A) Schematic of the Gea2-Arf1 activation intermediate complex used for cryoEM. (B) CryoEM density of the Gea2-Arf1 complex, colored and labeled as in Figure 1, with Arf1 colored purple. (C) Atomic model of the Gea2-Arf1 complex. (D) Views of the Gea2 GEF domain and GEF-HDS1 linker for each of the three conformations adopted by Gea2 in the Gea2 only (closed and open) and Arf1-bound conformations. See also Figures S5-S7.

To further investigate the role of the regulatory domains in modulating the action of the GEF domain, we trapped the Gea2-Arf1 nucleotide-free activation intermediate (Figure S1F) and determined its structure by cryoEM (Figures 4A-4C and S6; Table 1). The conformation of nucleotide-free Arf1 in our full-length Gea2-Arf1 complex structure was nearly identical to that of nucleotide-free Arf1 when bound to the isolated Gea2 GEF domain determined previously by X-ray crystallography (Figure S7A) (Goldberg, 1998). Strikingly, a closed conformation of the GEF domain was not observed in the Gea2-Arf1 complex cryoEM data; instead the position of the Arf1-bound GEF domain was similar to that of the open conformation observed in the absence of Arf1 (Figures 4D, S6, and S7B). We note that structural predictions of Gea2, its yeast paralog Gea1, and its human homolog GBF1 each adopt the closed conformation (Figure S7C). These structural results suggest that binding to nucleotide-free Arf1 enforces an open conformation of the Gea2 GEF domain.

**Figure 5.**
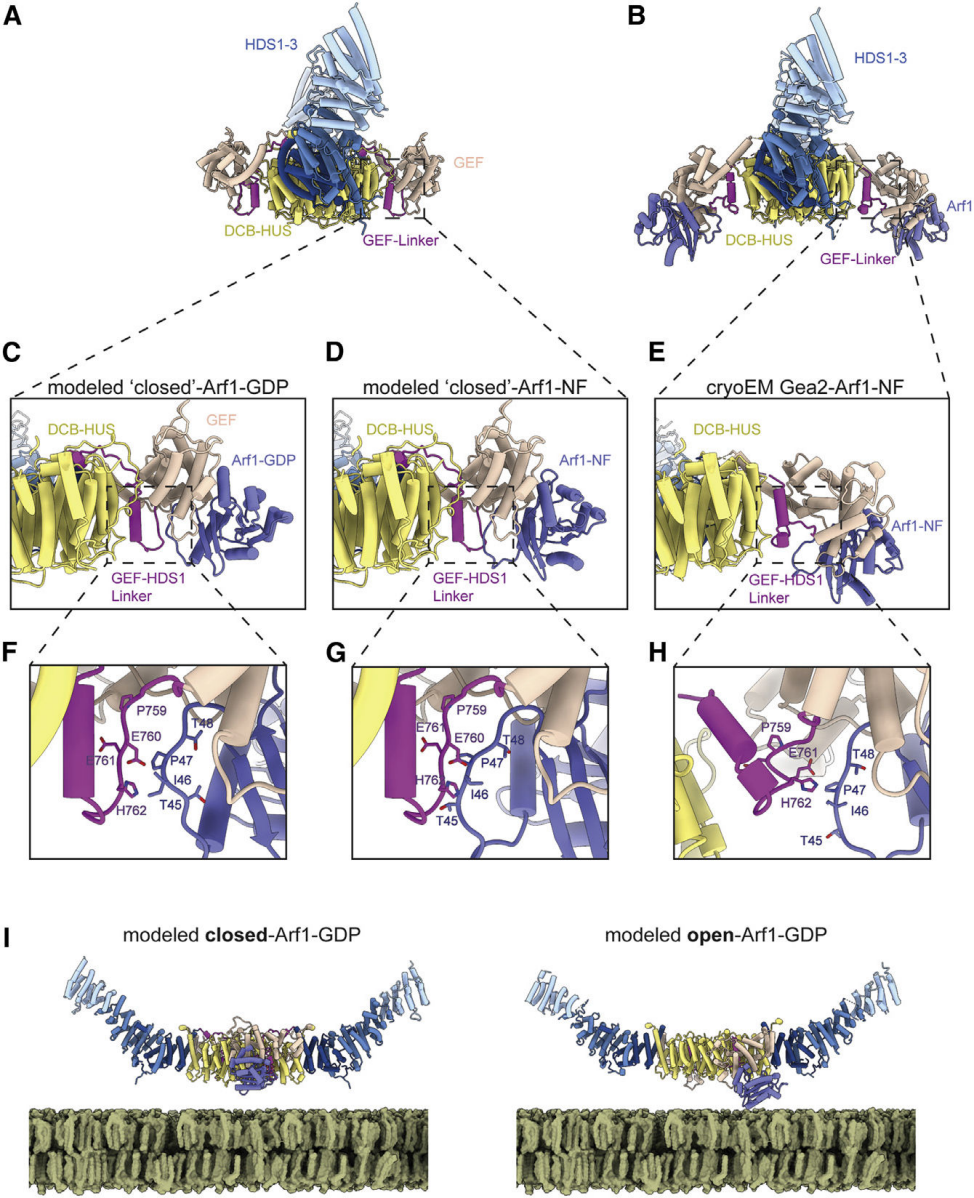
Steric constraints appear to enforce Gea2 conformational change (A) Structure of the closed/closed Gea2 dimer shown for context. (B) Structure of the Gea2-Arf1 complex shown for context. (C) Close-up view of the modeled Gea2 closed-Arf1-GDP complex. (D) Close-up view of the modeled Gea2 closed-Arf1-NF (nucleotide-free) complex. (E) Close-up view of the Gea2-Arf1-NF cryoEM structure. (F) Magnified view of (C). (G) Magnified view of (D). Note the steric clash between Arf1 and the GEF-HDS1 linker. (H) Magnified view of (E). (I) Comparison of the modeled closed/closed Gea2-Arf1-GDP complex with the modeled open/open Gea2-Arf1-GDP complex. Note how in the closed conformation, the GEF domain appears more readily able to encounter freely diffusing Arf1-GDP, compared with the open conformation.

The conserved GEF-HDS1 linker adopts distinct conformations when in the closed, open, and Arf1-bound states (Figures 4D, S5C, and S5D) and therefore appears important for stabilizing each of these states. In the closed conformation the entire GEF-HDS1 linker is ordered, whereas nearly 20 residues (residue numbers 781–798) at the C-terminal end of the GEF-HDS1 linker are disordered in the open and Arf1-bound structures. To understand why the closed conformation was not observed in the Arf1-bound complexes, we generated a series of models representing different stages of the established Arf1 activation pathway (Figures 5A-5H). To model nucleotide-free Arf1 bound to the closed conformation of Gea2, we superimposed our structure of nucleotide-free Arf1 bound to the GEF domain onto the GEF domain of the closed complex (Figure 5D). This modeled complex resulted in a steric clash between the “switch I” region of Arf1 and the GEF-HDS1 linker of Gea2 (Figure 5G). This indicates that the Gea2 closed conformation is incompatible with binding to the nucleotide-free state of Arf1. This steric clash with the closed conformation also explains why the nucleotide-free Gea2-Arf1 activation intermediate adopts an open conformation.

### Evidence for GEF conformational switching during Arf1 nucleotide exchange

These findings raised the question of whether the closed conformation served any role in the nucleotide exchange reaction. We therefore superimposed the published structure of Arf1-GDP bound to the GEF domain from ARNO (Renault et al., 2003) onto the closed conformation of Gea2 (Figure 5C). In contrast to the nucleotide-free state, Arf1-GDP appears able to bind to Gea2 in the closed conformation without clashes (Figure 5F), because the configuration of the Arf1 “switch I” region is different in the GDP-bound and nucleotide-free states. This suggests that the closed conformation of Gea2 is compatible with binding to Arf1-GDP.

We were initially puzzled by our observation that the “open” position of the GEF domain in the nucleotide-free Gea2-Arf1 complex appears unsuitable for the initial association event between Gea2 and Arf1-GDP, assuming Gea2 is already membrane bound. The orientation of the GEF domain active site facing toward the membrane suggested that its close proximity to the membrane would preclude it from productively encountering its substrate Arf1-GDP via diffusion, either from the cytosol or along the membrane surface. In contrast, the closed conformation, in which the GEF domain active site is oriented orthogonal to the membrane surface, appears much more suitable for productive encounters with the Arf1-GDP substrate via diffusion than does the open conformation (Figure 5I).

Taken together, our structural analysis suggests that initial binding to Arf1-GDP likely occurs with the Gea2 GEF domain in the closed conformation (Figures 5C and 5F). Subsequent release of GDP, triggered by interaction with the GEF domain, causes Arf1 to adopt its nucleotide-free structure. As this conformation of Arf1 is incompatible with the Gea2 closed state (Figures 5D and 5G), the GEF domain likely switches to the open state concurrent with nucleotide release, adopting the nucleotide-free Arf1-bound conformation we observed by cryoEM (Figures 5E and 5H). Given the apparent independence of each GEF domain in the dimer, it is also possible that only one GEF domain is able to adopt the open conformation at a time when Gea2 is bound to the membrane. This possibility would enable the Gea2 dimer to remain more closely associated with the membrane surface throughout the activation reaction.

### A model for activation-coupled membrane insertion of Arf1

When bound to Gea2 in its nucleotide-free state, Arf1 is positioned such that its N terminus is oriented toward the membrane surface, and we predict it to be in close proximity to the lipid headgroups (Figure 5I). Although not present in the construct we used to determine the structure of the complex, the N terminus of Arf1 folds into a membrane-inserting amphipathic helix upon GTP binding (Antonny et al., 1997; Liu et al., 2010). The conformation of Gea2 when bound to the nucleotide-free intermediate therefore appears to prime Arf1 for membrane insertion: GTP binding to the nucleotide-free intermediate induces formation of the N-terminal Arf1 amphipathic helix in a position optimal for its insertion into the cytoplasmic leaflet of the Golgi membrane.

**Figure 6.**
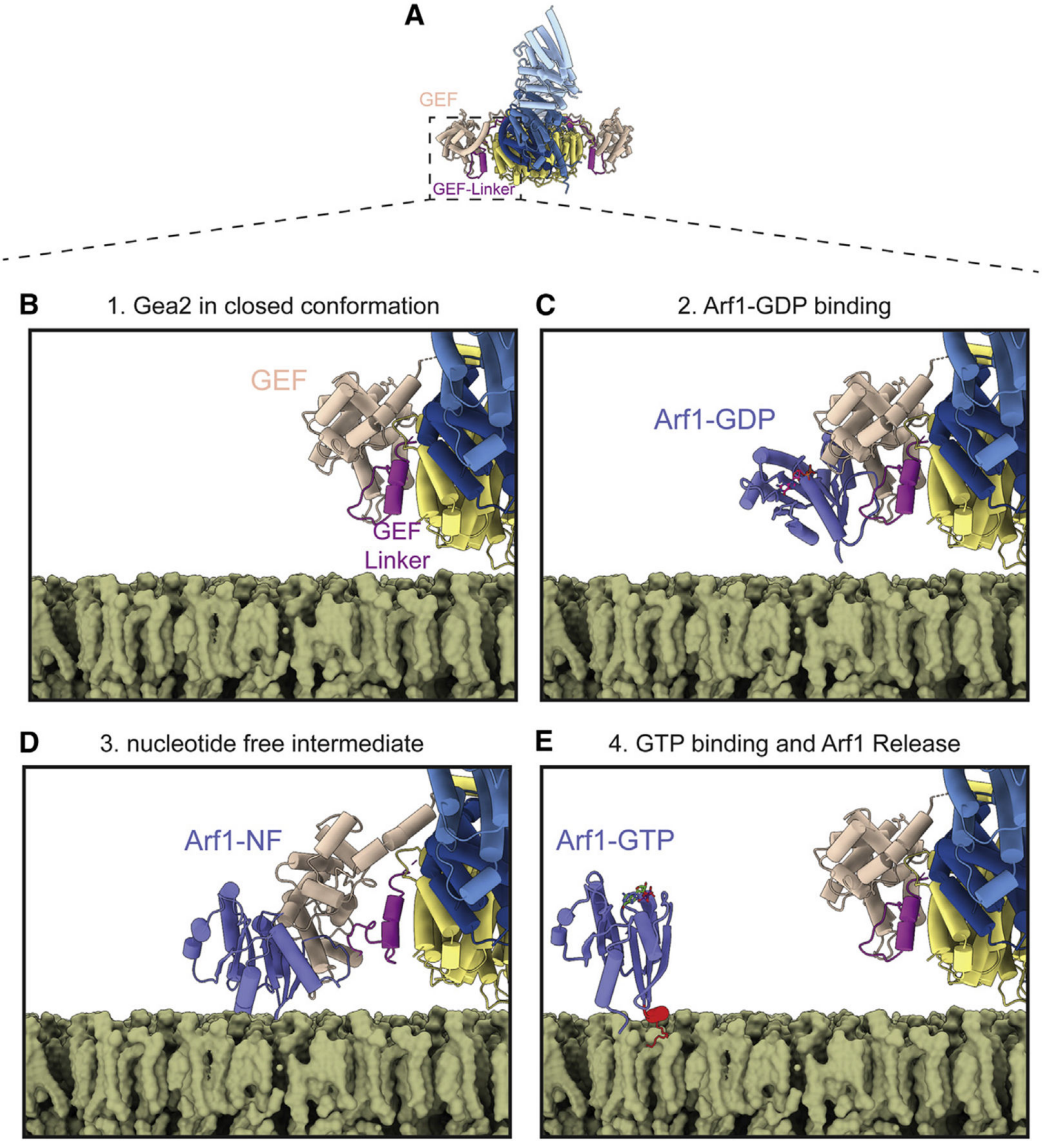
Model for activation of Arf1 by Gea2 on the Golgi membrane surface (A) Gea2 in the closed/closed conformation shown for context. (B) In step 1, at least one of the Gea2 monomers adopts the closed conformation while bound to the membrane surface (the cryoEM structure of one side of the closed/closed conformation is shown on a modeled membrane). (C) In step 2, Arf1-GDP binds to the GEF domain (the modeled closed-Arf1-GDP complex is shown). (D) In step 3,GDP dissociates from Arf1 (Arf1-NF = nucleotide-free), and the resulting conformation change in Arf1 causes the GEF domain to switch from the closed state to an open state in order to avoid steric clash with Arf1 (the Gea2-Arf1 cryoEM structure is shown). (E) In step 4, GTP binding causes another conformation change in Arf1, resulting in folding of its amphipathic helix (colored red) at the membrane surface and dissociation from Gea2 (the NMR structure of Arf1-GTP and cryoEM structure of the closed/closed conformation of Gea2 are shown). The structures of Arf1-GDP and Arf1-GTP were derived from RCSB entries PDB: 1R8S (Renault et al., 2003) and 2KSQ (Liu et al., 2010). See also Video S1.

Our structural results and analyses lead us to a complete model for nucleotide exchange-coupled membrane insertion of Arf1 by Gea2 (Figure 6 and Video S1). Arf1-GDP initially encounters membrane-bound Gea2 in its closed conformation (Figures 6A-6C). Nucleotide release then leads to an open conformation to avoid steric clash with the GEF-HDS1 linker. The resulting open conformation positions the N terminus of Arf1 optimally for membrane insertion (Figure 6D). Finally, GTP binding triggers membrane insertion of Arf1 via folding of its myristoylated amphipathic helix and release from Gea2 (Figure 6E).

## DISCUSSION

Arf1 is known for its role as a regulator of the function and regulation of the Golgi complex and recycling endosomes, but its activity has also been implicated in endocytosis, TORC1 kinase signaling, lipid droplet homeostasis, and lysosomal and mitochondrial function (Ackema et al., 2014; Dechant et al., 2014; Kumari and Mayor, 2008; Su et al., 2020; Wilfling et al., 2014). A hallmark of Ras-related “small” GTPases such as Arf1 is the structural transitions they undergo during nucleotide exchange and hydrolysis. Arf1 is the founding member of the Arf GTPase family, which includes more than 20 proteins in humans which collectively regulate virtually all membrane trafficking pathways (Gillingham and Munro, 2007). Most Arf family GTPases are anchored to the membranes of organelles and vesicles by their N-terminal amphipathic helices. Unlike other Ras-related GTPases, when inactive these membrane-anchoring motifs are masked by direct interaction with the GDP-bound Arf1 nucleotide-binding domain (Amor et al., 1994). In contrast, Rab and Rho family GTPases employ chaperone proteins (guanine nucleotide displacement inhibitors) to mask their membrane-anchoring motifs in the GDP-bound state (Isomura et al., 1991; Soldati et al., 1994). GTP binding exposes the Arf amphipathic helix, inducing stable membrane binding (Antonny et al., 1997). Although membrane insertion of GTP-bound Arf proteins is favorable, there is likely a kinetic “activation energy” barrier that slows the membrane-insertion step, as it requires lipids to rearrange in order to accommodate the amphipathic helix. Our structural findings point to a mechanism for how Gea2 may reduce this kinetic barrier by positioning Arf1 optimally for membrane insertion.

To our knowledge, conformational change of a GEF during the nucleotide exchange reaction has not been reported. Several GEFs are known to be autoinhibited and/or allosterically activated, and the structural basis for autoinhibition and activation has been documented for several GEFs, including the Ras-GEF SOS (Gureasko et al., 2008; Sondermann et al., 2004), the Rab-GEF Rabex5 (Delprato and Lambright, 2007; Lauer et al., 2019; Zhang et al., 2014), the Arf-GEF Cytohesin/Grp1 (Das et al., 2019; DiNitto et al., 2007; Malaby et al., 2013), and the Rho-GEF Vav (Yu et al., 2010). In the context of autoinhibition and allosteric activation, GEF conformational change is usually coupled to phosphorylation or binding to a regulatory protein or lipid and is a prerequisite for the nucleotide exchange reaction. In contrast, Gea2 appears to capitalize on the conformational changes its substrate GTPase undergoes during nucleotide exchange to drive its own conformational change during the activation reaction. It is also possible that the transition of Gea2 to the open state may provide an additional driving force for nucleotide release.

A mutation has been identified in geaA, the *Aspergillus nidulans* homolog of *S. cerevisiae* Gea2, corresponding to a Y1001C substitution in Gea2 that partially suppressed the loss of the *A. nidulans* homolog of Sec7, hypB (Arst et al., 2014). Remarkably, this Y-to-C substitution mutation shifted the localization of geaA from early Golgi compartments toward later Golgi compartments normally occupied by hypB. Our findings provide a mechanistic interpretation of this observation, as we have identified Y1001 as a critical residue for Gea2 membrane interaction through our use of the Y1001D mutant. An interesting possibility is that the Y-to-C substitution, by modulating but not eliminating the hydrophobicity of the amphipathic helix, alters which membranes are most favored for stable binding due to their compositions or biophysical properties. We note that in contrast to the results reported for *A. nidulans* geaA, we found that the equivalent Y-to-C substitution did not enable Gea2 to suppress loss of Sec7 in *S. cerevisiae* (Gustafson, 2017). This highlights the proposed roles of regulatory protein-protein interactions in directing the localization of the Golgi Arf-GEFs to specific compartments (Christis and Munro, 2012; Gustafson and Fromme, 2017; Lowery et al., 2013; McDonold and Fromme, 2014; Monetta et al., 2007; Richardson et al., 2012).

There are likely to be both similarities among and differences between the structural mechanisms underlying Arf1 activation by Gea2 and Sec7. Previous work on Sec7 highlighted the influence of the DCB-HUS domain on the activity of the GEF domain for activation of Arf1 on the membrane surface (Halaby and Fromme, 2018; Richardson et al., 2016). However, Sec7 likely adopts a very different overall architecture because Sec7 dimerizes via its HDS4 domain (Richardson et al., 2016). Sec7 is also regulated by distinct positive feedback, autoinhibition, and crosstalk mechanisms (McDonold and Fromme, 2014; Richardson et al., 2012) and prefers more anionic membranes compared with Gea1/Gea2 (Gustafson and Fromme, 2017).

Although we have now identified how Gea2 interacts with membranes, how it achieves its specific localization remains unresolved. Both Gea1 and Gea2, as well as GBF1, interact with Rab1/Ypt1, which likely recruit these Arf-GEFs to the Golgi, yet Gea1 and Gea2 localize to distinct Golgi compartments (Gustafson and Fromme, 2017; Monetta et al., 2007). Future studies are required to characterize the Gea2-Rab1/Ypt1 interaction and determine how Gea1 and Gea2 achieve their specific localization.

### Limitations of the study

The structural data support a role for GEF domain conformational change in coupling Arf1 activation with membrane insertion, but in-depth experimental validation is required to fully test this hypothesis. Further study is also required to characterize additional aspects of the membrane-proximal activation mechanism. Important mechanistic questions include the precise timing of when the Arf1 amphipathic helix inserts into the membrane during the activation reaction and whether the two GEF domains can perform the activation reaction simultaneously.

The resolution of the cryoEM maps enabled us to confidently fit side chains for virtually all of the modeled residues, but there are a small number of residues for which it is formally possible that our amino acid assignments may be incorrect. For example, for a portion of the GEF-HDS1 linker in the open conformation, the cryoEM map density of some side chains is not well resolved. Fortunately, comparison with the corresponding more clearly resolved cryoEM map density of the closed and Arf1-bound conformations was helpful in this case, and any imprecision in residue assignment of this region is not expected to impact the interpretations and conclusions made in this study.

## STAR★METHODS

### RESOURCE AVAILABILITY

#### Lead contact

Further information and requests for materials should be directed to and will be fulfilled by the lead contact, J. Christopher Fromme (jcf14@cornell.edu).

#### Materials availability

Plasmids and strains generated in this study will be provided by the lead contact upon request.

#### Data and code availability

- Atomic coordinates and cryoEM density maps have been deposited in the Protein DataBank (RCSB PDB) and in the Electron Microscopy DataBank (EMDB). Accession numbers are listed in the key resources table.
- This paper does not report original code.
- Any additional information required to reanalyze the data reported in this paper is available from the lead contact upon request.

### EXPERIMENTAL MODEL AND SUBJECT DETAILS

All recombinant plasmids and yeast strains were generated using standard molecular biology techniques and are listed in the key resources table. Plasmids were constructed using the DH5α strain of *E. coli* (New England Biolabs). Arf1 constructs were purified from the Rosetta2 strain of *E. coli* (Novagen). *E. coli* strains were cultured in LB and TB media. Yeast cell viability assays and yeast cell imaging was performed as described below using *S. cerevisiae* strains listed in the key resources table. *S. cerevisiae* was cultured in standard yeast synthetic dropout media. *P. pastoris* strains used for expression of Gea2 constructs were cultured in BMGY media and are listed in the key resources table.

### METHOD DETAILS

#### Protein purifications

Full-length *S. cerevisiae* Gea2 was cloned with an N-terminal cleavable 6xHis-tag into the pPICZ vector, then purified using *Pichia pastoris.* An overnight culture of “BMGY” media was used to inoculate a 200mL BMGY starter culture. After 8 h of shaking at 30°C, 120 mL of this starter culture was used to inoculate 6 liters of “autoinduction media” (Lee et al., 2017) and then shaken overnight at 30°C. After overnight growth, additional methanol was added (equivalent to additional 0.5% final concentration) and the cultures were shaken for an additional 24 h at 30°C. Cells were harvested by centrifugation (2000 g, 10 min), resuspended in lysis buffer (50 mM Tris pH 8.0, 500 mM NaCl, 10% glycerol, 20 mM imidazole 10 mM βME), and lysed under liquid nitrogen using an SPEX 6875D freezer mill. Lysed cells were cleared using centrifugation (40,000 g, 1 h) and the supernatant was incubated with 1 mL Ni^2+^-NTA resin for 1 h. Resin was washed with lysis buffer and the protein was eluted with elution buffer (50 mM Tris pH 8.0, 500 mM NaCl, 10% glycerol, 500 mM imidazole, 10 mM BME). The elute was then diluted 5x with Buffer A (20 mM Tris pH 8.0, 1 mM DTT) and subjected to ion exchange using a MonoQ column (Buffer B = Buffer A + 1 M NaCl). Fractions were visualized by SDS page and pooled fractions were concentrated to 500 μL total volume then treated with 50 μL of 1 mg/mL TEV protease overnight at 4°C. The sample was further purified by size exclusion chromatography using a Superdex 200 Increase column equilibrated in SEC buffer (20 mM Tris pH 8.0, 150 mM NaCl, 1 mM DTT). The Y1001D mutant was purified using the same procedure.

*S. cerevisiae* ΔN17-Arf1 and myristoylated-Arf1 were purified as previously described (Richardson and Fromme, 2015; Richardson et al., 2012).

#### Gea2-Arf1 complex formation

The Gea2-Arf1 complex was prepared by incubating 1 mg of Gea2, 5 mg ΔN17-Arf1, and 250 units alkaline phosphatase in 1.5 mL reaction volume at 4°C overnight. The complex was then purified by size exclusion chromatography using a Superdex 200 Increase column equilibrated in SEC buffer (20 mM Tris pH 8.0, 300 mM NaCl, 1 mM DTT).

#### CryoEM sample preparation and data collection

3.5 μL of Gea2 or the Gea2-Arf1 complex, at ~5 mg/mL in SEC Buffer containing 2 mM fluorinated fos-choline-8 (Anatrace, cat# F300F), was applied to glow discharged Quantifoil R1.2/1.3 grids, blotted for 5 s, then plunge-frozen into liquid ethane using a Vitro-bot Mark IV. Imaging was done at 63kX nominal magnification on a Talos Arctica operating at 200kV equipped with a K3 detector and BioQuantum energy filter. For Gea2 alone, ~8,000 movies were collected over multiple sessions, and for the Gea2-Arf1 complex ~2500 movies were collected. Movie exposures were collected using SerialEM (Mastronarde, 2005) using the multi-shot feature with coma correction. All data was collected using 100 frames per movie exposure with a total dose of ~50 e^−^/Å^2^.

#### CryoEM data processing

##### Gea2 alone

Movie exposures were motion-corrected and dose-corrected using MotionCor2 (Zheng et al., 2017). Corrected micrographs were imported into cryoSPARC (Punjani et al., 2017) and then subjected to patch-CTF estimation. Particle picking was performed via TOPAZ (Bepler et al., 2019, 2020) using a ‘general’ model. Picked particles were parsed with 2D classification and rounds of 3D classification (see Figure S1). A clean particle stack was generated and imported into RELION 3.1 (Zivanov et al., 2018, 2020) and particles were 3D classified revealing three distinct conformations. Particles in each of these three major classes were kept separate for the rest of the processing steps. Particles were subjected to multiple rounds of CTF refinement and Bayesian polishing (Zivanov et al., 2019). C2 symmetry was enforced during refinements of the open and closed states. After the iterative refinement process converged, particles from the closed/closed and open/open states were symmetry expanded and signal subtracted using a monomer mask (Nakane et al., 2018). For the closed/open state, an additional refinement was performed with C2 symmetry enforced in order to perform symmetry expansion and monomer particle subtraction. 3D classification was then used to generate separated particle stacks for the open and closed monomers. Following monomer refinements, subsequent signal subtraction and local refinements were performed separately on the N and C terminal regions. An additional signal subtraction and focused refinement was performed for the dimer interface of each of the three states (open, closed, and hemi). Density modification (Terwilliger et al., 2020) was then used to further improve all of the focused maps. Composite maps used for model building and refinement of each of the three dimeric conformations were generated with ‘Combine Focused Maps’ in Phenix (Liebschner et al., 2019). See Figures S2, S3, and Table 1.

##### Gea2-Arf1 complex

The cryoEM data collected for the Gea2-Arf1 complex was processed using the same procedure described above for Gea2 alone. 3D classification indicated that the sample was conformationally homogeneous, adopting a single conformation. After symmetry expansion and signal subtraction, focused refinements were performed on the DCB-HUS, GEF, and HDS1-3 regions. Density modification (Terwilliger et al., 2020) was used to further improve all of the focused maps, and composite maps used for model building and refinement were generated with ‘Combine Focused Maps’ in Phenix (Liebschner et al., 2019). See Figure S6 and Table 1.

#### Atomic model building and refinement

The composite maps described above were used for atomic model building and refinement. Model building in Coot (Emsley et al., 2010) was guided by the AlphaFold prediction of Gea2 (Jumper et al., 2021) and by the Gea2 GEF domain - Arf1 crystal structure (Goldberg, 1998). Real space refinement and model validation was carried out using Phenix (Afonine et al., 2018; Emsley et al., 2010). See Figures S3 and S6 and Table 1.

#### Yeast complementation assay

Gea2-expressing yeast plasmids were transformed into a Gea1/2 yeast shuffling strain (*gea1*Δ *gea2*Δ strain CFY2872) and grown overnight at 30°C. Cultures were normalized by OD600 and serial diluted on selection media. Plates were then incubated for three days at 30°C before imaging.

#### Fluorescence microscopy

Gea2-expressing yeast plasmids were transformed into *gea2*Δ yeast strain (CFY1470) and grown at 30°C in selection media to an OD600 of 0.6. Cells were added to an imaging dish (MatTek), allowed to settle for 10 min, then washed with fresh media. Cells were imaged using a CSU-X spinning-disk confocal system (Intelligent Imaging Innovations) with a DMI6000 B microscope (Leica), 100×1.46 NA oil immersion objective, and a QuantME EMCCD camera (Photometrics), using with a 200 μs exposure time.

#### Liposome preparation

Liposomes were prepared as reported previously (Richardson and Fromme, 2015) and described here: lipid mixes in choloroform, with lipid compositions described further below, were vacuum-dried in pear-shaped flasks using a rotary evaporator and then rehydrated overnight at 37°C in HK buffer (20mM HEPES pH 7.5, 150 mM KOAc). The resulting liposomes were extruded through 100 nm filters for GEF assays or 400 nm filters for membrane-binding assays. Liposomes were extruded using 19 passes through the filter and stored at 4°C.

#### *In vitro* membrane-binding assay

Liposome pelleting assays were performed as reported previously (Gustafson and Fromme, 2017; Paczkowski and Fromme, 2016) and described here: Liposomes were prepared as described above using a lipid mix consisting of 94% DOPC, 5% Nickel-DOGS, and 1% DiR lipids. 500 μg liposomes were incubated with 8 ug of protein in 50 μL total reaction volume in HK buffer for 10 min at room temperature. Reactions were then subjected to ultracentrifugation (128,000 g for 10 min). The supernatant was separated and the liposome pellet was resuspended in HK buffer. Supernatant and pellet samples were analyzed by SDS-PAGE.

#### *In vitro* GEF activity assay

GEF activity assays were performed as reported previously (Gustafson and Fromme, 2017; Richardson and Fromme, 2015) and described here: Liposomes were prepared as described above using a lipid mix consisting of 99% DOPC and 1% DiR lipids. All reactions were performed in HKM buffer (20mM HEPES pH 7.5, 150 mM KOAc, 1 mM MgCl_2_) at 30°C. The nucleotide-bound state of Arf1 was monitored in real-time by native tryptophan fluorescence (297.5 nm excitation, 340 nm emission). Myristoylated-Arf1 activation reactions were performed by incubating 333 μM liposomes, 200 nM Gea2, 200 μM GTP for 2 min before adding 1 μM myr-Arf1, and the change in fluorescence was then measured over time. These activation traces were fit to a single-exponential curve to determine the rate-constant ‘k’, and the experimental nucleotide exchange rates were calculated by dividing ‘k’ by the GEF concentration used in the reaction. ΔN17-Arf1 activation was assessed in similar reactions, except liposomes were omitted, Gea2 concentration was 25 nM, and ΔN17-Arf1 concentration was 500 nM.

### QUANTIFICATION AND STATISTICAL ANALYSIS

Statistical tests were performed using GraphPad Prism Software. For the data presented in Figures 3G-3I, error bars represent 95% confidence intervals for n = 3 technical replicates. For Figures 3G and 3I significance was assessed by Student’s T test, and the Figure 3 legend indicates the significance values.

## Supplementary Material

1

2

## Figures and Tables

**Table 1.** CryoEM data collection, processing, and model validation statistics

	Gea2 closed/ open (composite map)	Gea2 closed/ open (consensus map)	Gea2 closed/ closed (composite map)	Gea2 closed/ closed (consensus map)	Gea2 open/open (composite map)	Gea2 open/open (consensus map)	Gea2-Arf1 complex (composite map)	Gea2-Arf1 complex (consensus map)
Nominal magnification	63,000							
Voltage (kV)	200							
Total dose (e^−^/Å^2^)	50							
Defocus range (μm)	−1.0 to −2.0							
Pixel size (Å)	1.29						1.30	
Symmetry imposed	N/A	C1	N/A	C2	N/A	C2	N/A	C2
Particle images		100,455		87,338		78,176		391,360
Map resolution, 0.143-FSC (Å)^a^		4.4		4.1		4.2		4.2
Map sharpening	N/A	−72	N/A	−112	N/A	−99	N/A	−167
B factor								
No. of atoms	38,168	N/A	39,740	N/A	36,590	N/A	43,048	N/A
No. of protein residues	2345		2,448		2,242		2,650	
B factor, protein	174		149		144		129	
RMSD, bond length (Å)	0.006		0.006		0.006		0.006	
RMSD, bond angles (°)	0.962		0.933		0.977		0.996	
MolProbity score	1.66		1.63		1.60		1.64	
Clashscore	5.87		5.16		4.45		5.48	
Poor rotamers (%)	0.23		0.53		0.72		0.29	
Ramachandran plot								
Favored (%)	94.00		94.91		94.50		95.00	
Allowed (%)	5.87		5.05		5.32		5.00	
Disallowed (%)	0.13		0.04		0.18		0	

RMSD, root-mean-square deviation; N/A, not applicable.

a FSC curves for focused and consensus maps are presented in Figures S3 and S6.

**Table:** KEY RESOURCES TABLE

REAGENT or RESOURCE	SOURCE	IDENTIFIER
Antibodies		
Mouse anti-GFP	Roche	Cat # 11814460001; RRID: AB_390913
Mouse anti-Pgk1	Molecular Probes	Cat # 22C5D8; RRID: AB_2532235
Bacterial and virus strains		
Rosetta2, *E. coli*	Novagen	Cat # 71400
DH5α, *E. coli*	New England Biolabs	Cat # C2987I
Chemicals, peptides, and recombinant proteins		
GTP	Thermo Fisher	Cat # R0461
Calf intestinal alkaline phosphatase	Sigma	Cat # P4978
1,2-dioleoyl-sn-glycero-3-phosphocholine (DOPC)	Avanti Polar Lipids	Cat # 850375
1,2-dioleoyl-sn-glycero-3-[(N-(5-amino-1-carboxypentyl)iminodiacetic acid)succinyl] (nickel salt) (Nickel-DOGS)	Avanti Polar Lipids	Cat # 790404
1,1’-Dioctadecyl-3,3,3’,3’-Tetramethylindotricarbocyanine Iodide (DiR’; DilC18(7))	Thermo Fisher	Cat# D12731
Fos-choline-8, fluorinated	Anatrace	Cat # F300F
Deposited data		
ΔN17-Arf1-GDP crystal structure	Renault et al. (2003)	PDB: 1R8S
Myristoylated-Arf1-GTP NMR structure	Liu et al. (2010)	PDB: 2KSQ
Gea2-Arf1 complex model	This paper	PDB:7URO
closed/open model	This paper	PDB:7URR
closed/closed model	This paper	PDB:7UT4
open/open model	This paper	PDB:7UTH
Gea2-Arf1 complex composite map	This paper	EMD-26716
closed/open composite map	This paper	EMD-26717
closed/closed composite map	This paper	EMD-26754
open/open composite map	This paper	EMD-26770
Gea2-Arf1 complex consensus map	This paper	EMD-26749
Gea2-Arf1 complex DCB-HUS domains focused map	This paper	EMD-26750
Gea2-Arf1 complex GEF domain-Arf1 focused map	This paper	EMD-26751
Gea2-Arf1 complex HDS domains focused map	This paper	EMD-26752
Gea2-Arf1 complex dimer interface focused map	This paper	EMD-26753
closed/closed consensus map	This paper	EMD-26755
closed/closed DCB-HUS domains focused map	This paper	EMD-26765
closed/closed GEF domain focused map	This paper	EMD-26766
closed/closed HDS domains focused map	This paper	EMD-26769
closed/closed dimer interface focused map	This paper	EMD-26777
open/open consensus map	This paper	EMD-26771
open/open DCB-HUS domains focused map	This paper	EMD-26773
open/open GEF domain focused map	This paper	EMD-26774
open/open HDS domains focused map	This paper	EMD-26775
open/open dimer interface focused map	This paper	EMD-26776
closed/open consensus map	This paper	EMD-26797
closed/open closed monomer DCB-HUS domains focused map	This paper	EMD-26779
closed/open closed monomer GEF domain focused map	This paper	EMD-26780
closed/open closed monomer HDS domains focused map	This paper	EMD-26781
closed/open open monomer DCB-HUS domains focused map	This paper	EMD-26783
closed/open open monomer GEF domain focused map	This paper	EMD-26784
closed/open open monomer HDS domains focused map	This paper	EMD-26785
closed/open dimer interface focused map	This paper	EMD-26778
Experimental models: Organisms/strains		
*MATα suc2-Δ9 ura3-52 his3-Δ200 leu2-3,112 lys2-801 trp1-Δ901*	Robinson et al. (1988)	SEY6210
*BY4741α gea1*Δ::*KanMX gea2*Δ::*HIS3* +pCF1248	Gustafson and Fromme (2017)	CFY2872
SEY6210 *gea2*Δ::*KanMX*	This study	CFY1470
KM71H (*P. pastoris*)	ThermoFisher Scientific	Cat # C18200
KM71H *pAOX1*::6xHis-TEV-Gea2::*BleoR*	This paper	CFY3882
KM71H *pAOX1*::6xHis-TEV-Gea2 (Y1001D)::*BleoR*	This paper	CFY4619
Recombinant DNA		
pPICZ: *P. pastoris* integration plasmid	ThermoFisher Scientific	Cat # V19020
pRS416-*GEA2*	Gustafson and Fromme (2017)	pCF1248
pRS415-Gea2-GFP	Gustafson and Fromme (2017)	pMG001
pRS415-Gea2(Δ996-1004)-GFP	This paper	pAM043
pRS415-Gea2(Y1001D)-GFP	This paper	pAM045
pPICZ-6xHis-TEV-Gea2	This paper	pAM034
pPICZ-6xHis-TEV-Gea2(Y1001D)	This paper	pAM050
pCF1053: ΔN17-Arf1	Richardson et al. (2012),	pCF1053
expression plasmid	Richardson and Fromme (2015)	
Full-length Arf1 expression plasmid	Weiss et al. (1989)	pArf1
Nmt1 expression	Duronio et al. (1990)	pNmt1
Software and algorithms		
SerialEM	Mastronarde (2005)	https://bio3d.colorado.edu/SerialEM/
MotionCor2	Zheng et al. (2017)	https://emcore.ucsf.edu/ucsf-software
CryoSPARC	Punjani et al. (2017)	https://cryosparc.com
RELION	Zivanov et al. (2018), Zivanov et al. (2020)	https://relion.readthedocs.io/en/release-3.1/
Phenix	Liebschner et al. (2019)	https://www.phenix-online.org/documentation/index.html
Coot	Emsley et al. (2010)	http://www2.mrc-lmb.cam.ac.uk/personal/pemsley/coot
TOPAZ	Bepler et al. (2019), Bepler et al. (2020)	https://cb.csail.mit.edu/cb/topaz/
SBGRID	Morin et al., 2013	https://sbgrid.org
Prism	GraphPad	https://www.graphpad.com/scientific-software/prism/
FIJI/ImageJ	Schindelin et al. (2012)	https://imagej.net/Fiji
Slidebook	Intelligent Imaging Innovations	https://www.intelligent-imaging.com/slidebook
Other		
Holey Carbon Grids, R 1.2/1.3, Au 300 mesh	Quantifoil	Cat # N1-C14nAu30-01
MonoQ 5/50 GL	GE Healthcare	Cat # 17516601
Ni-NTA Agarose Resin	Qiagen	Cat # 30210
Superdex 200 Increase 10/300	GE Healthcare	Cat # 28990944

## Highlights

- Multiple cryoEM structures of Gea2, including a Gea2-Arf1 activation intermediate
- Gea2 uses a conserved amphipathic helix for membrane binding
- The GEF domain adopts open and closed states, Arf1 binding enforces an open state
- GEF conformational change appears to couple Arf1 activation to membrane insertion
