# Supplementary material for: Structural basis for activation of Arf1 at the Golgi complex
Source: Cell Rep. Author manuscript; Available in PMC 2022 Sep 13. (PMC9469209; doi:10.1016/j.celrep.2022.111282)
Supplement: 1 [file NIHMS1833563-supplement-1.pdf]

Cell Reports, Volume *40*

Supplemental information

Structural basis for activation of Arf1 at the Golgi complex

Arnold J. Muccini, Margaret A. Gustafson, and J. Christopher Fromme

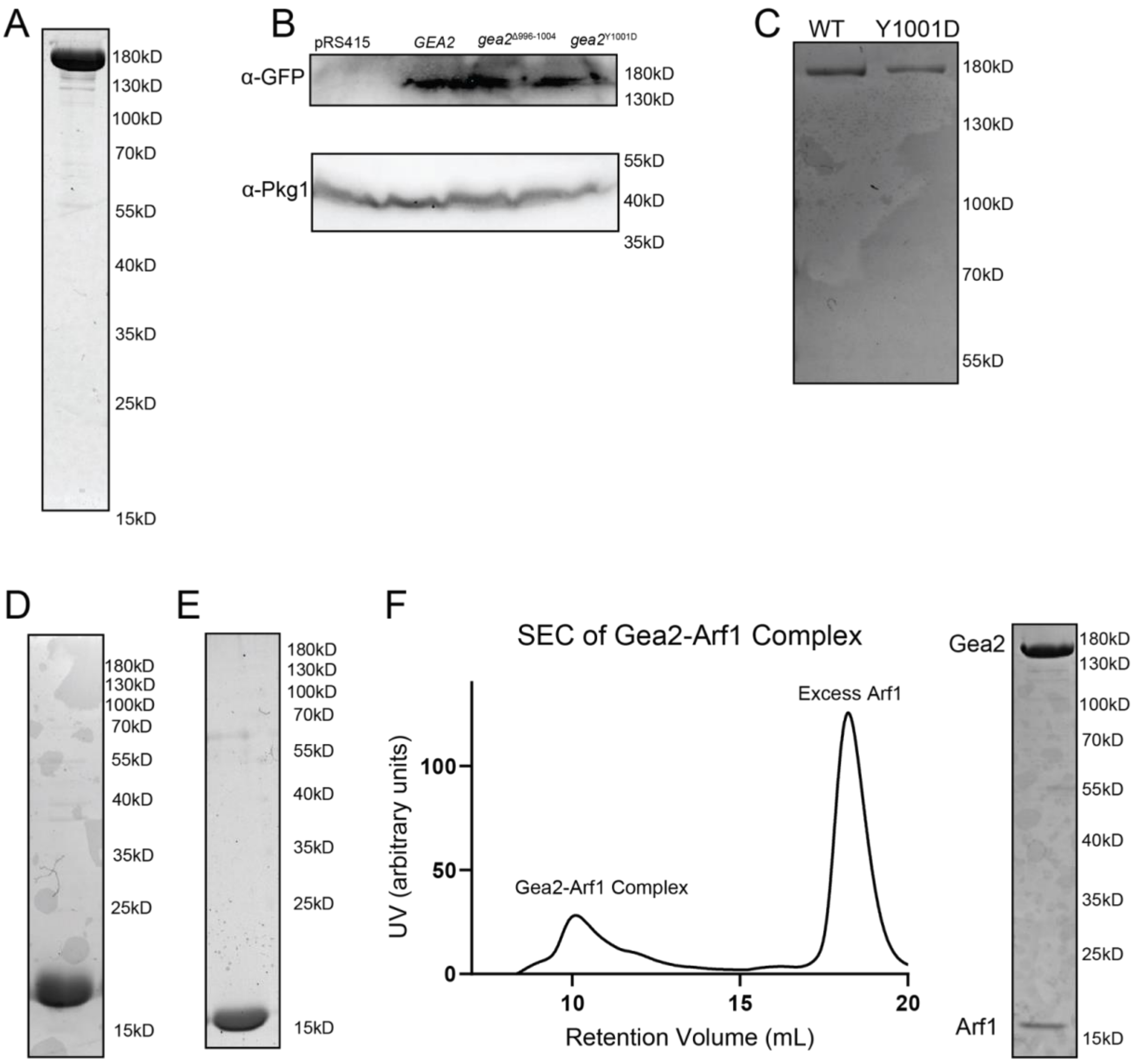

Figure S1. Protein reagents used in this study, Related to Figures 1, 3, and 4
A) SDS-PAGE analysis of *S. cerevisiae* Gea2 purified from *P. pastoris.* B) Immunoblot showing expression of Gea2-GFP constructs used in yeast localization experiments. C) SDS-PAGE analysis of wild-type and Y1001D mutant *S. cerevisiae* Gea2 proteins purified from *P. pastoris.* D) SDS-PAGE analysis of Myristoylated, full-length *S. cerevisiae* Arf1 purified from *E. coli*. E) SDS-PAGE analysis of ΔN17-mutant *S. cerevisiae* Arf1 purified from *E. coli*. F) Gel filtration chromatography trace and SDS-PAGE analysis of the Gea2-Arf1 activation intermediate complex.

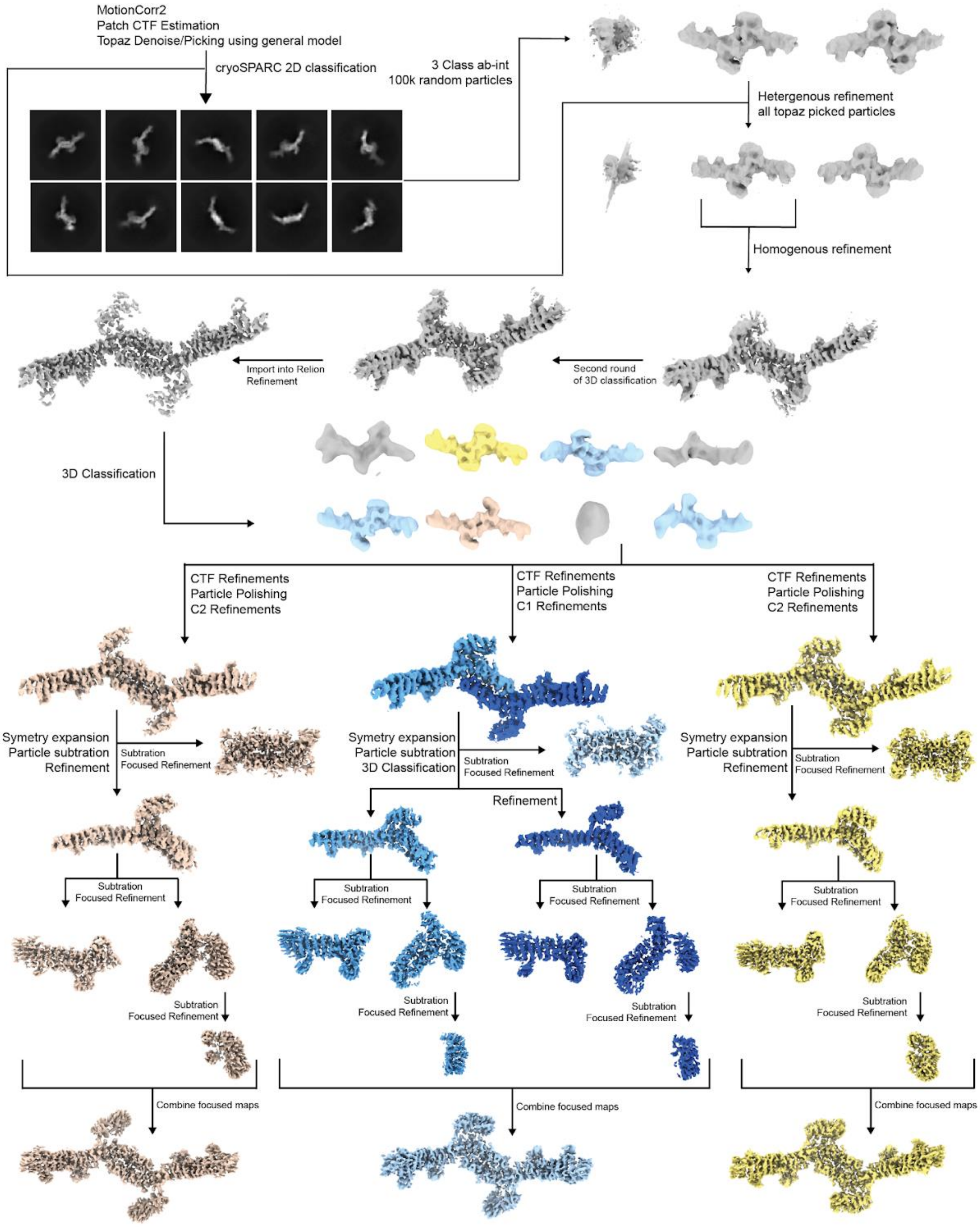

Figure S2. Gea2 cryoEM data processing, Related to Figure 1 and STAR Methods
Flowchart illustrating the data processing strategy for the Gea2 cryoEM data (see STAR Methods).

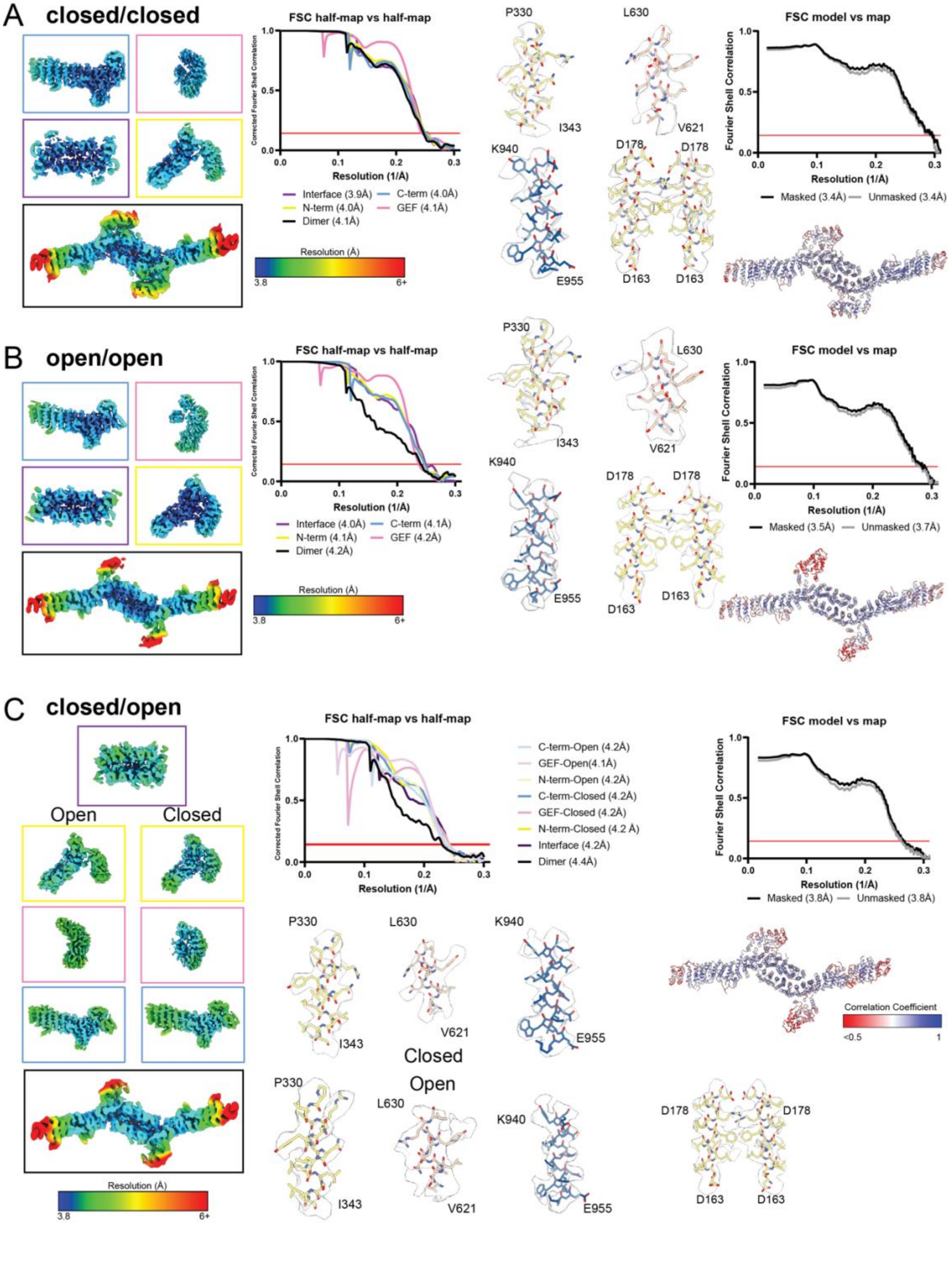

Figure S3. Gea2 cryoEM map and model validation, Related to Figure 1
A) Fourier shell correlation plots and example cryoEM density for focused refinements are shown for the cryoEM map and model of the Gea2 closed/closed conformation. B) Same but for the open/open conformation. C) Same but for the closed/open conformation. Note that the open conformation of the GEF domain exhibits more flexibility compared to the closed conformation.

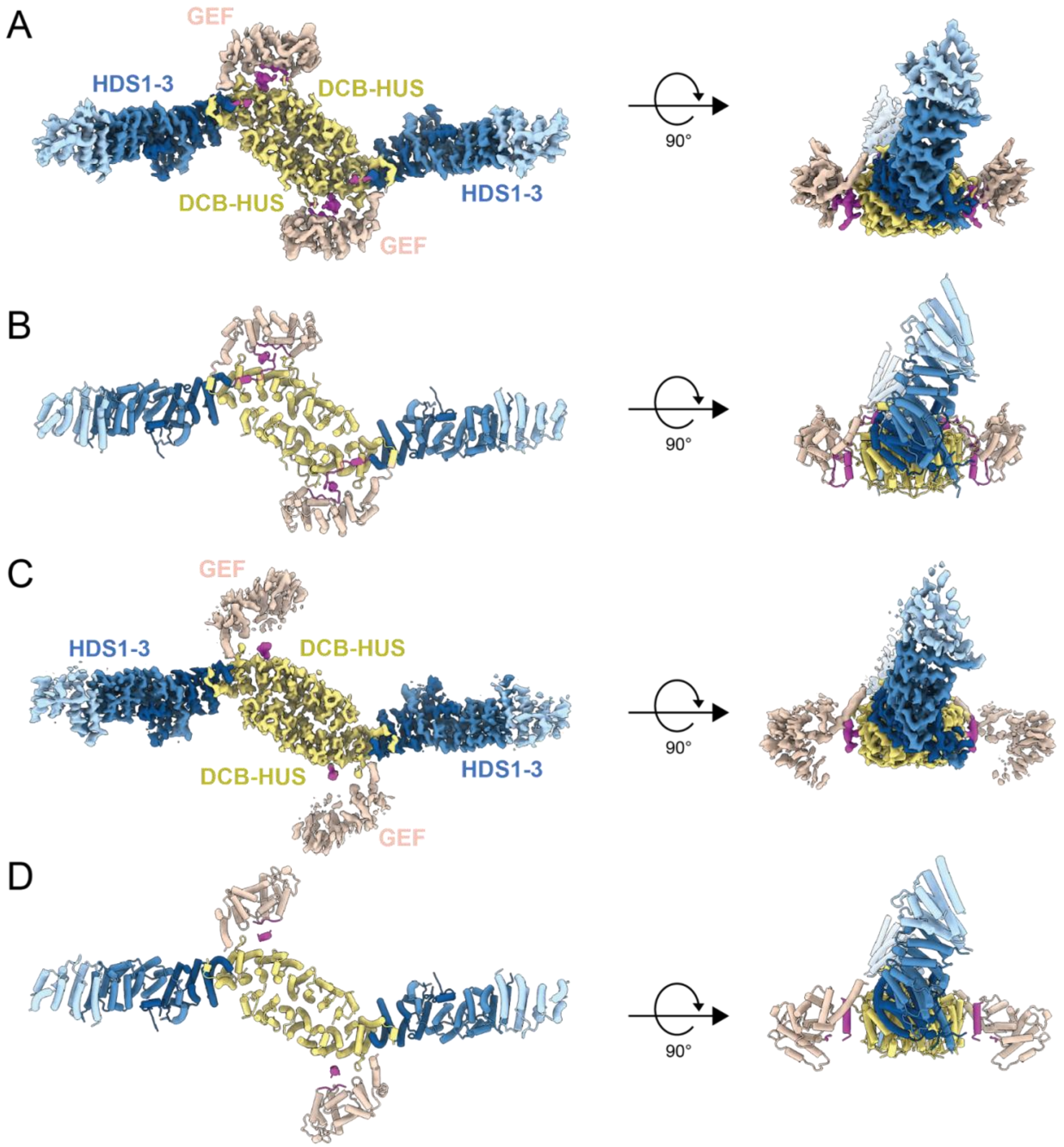

Figure S4. The closed/closed and open/open conformations of Gea2, Related to Figure 1
A) CryoEM density of the Gea2 dimer in its closed/closed conformation. Coloring as in Figure 1, the GEF-HDS1 linker is colored magenta. B) Atomic model of the Gea2 closed/closed dimer, shown in cartoon depiction. C) CryoEM density of the Gea2 dimer in its open/open conformation. Coloring as in Figure 1, the GEF-HDS1 linker is colored magenta. D) Atomic model of the Gea2 open/open dimer, shown in cartoon depiction.

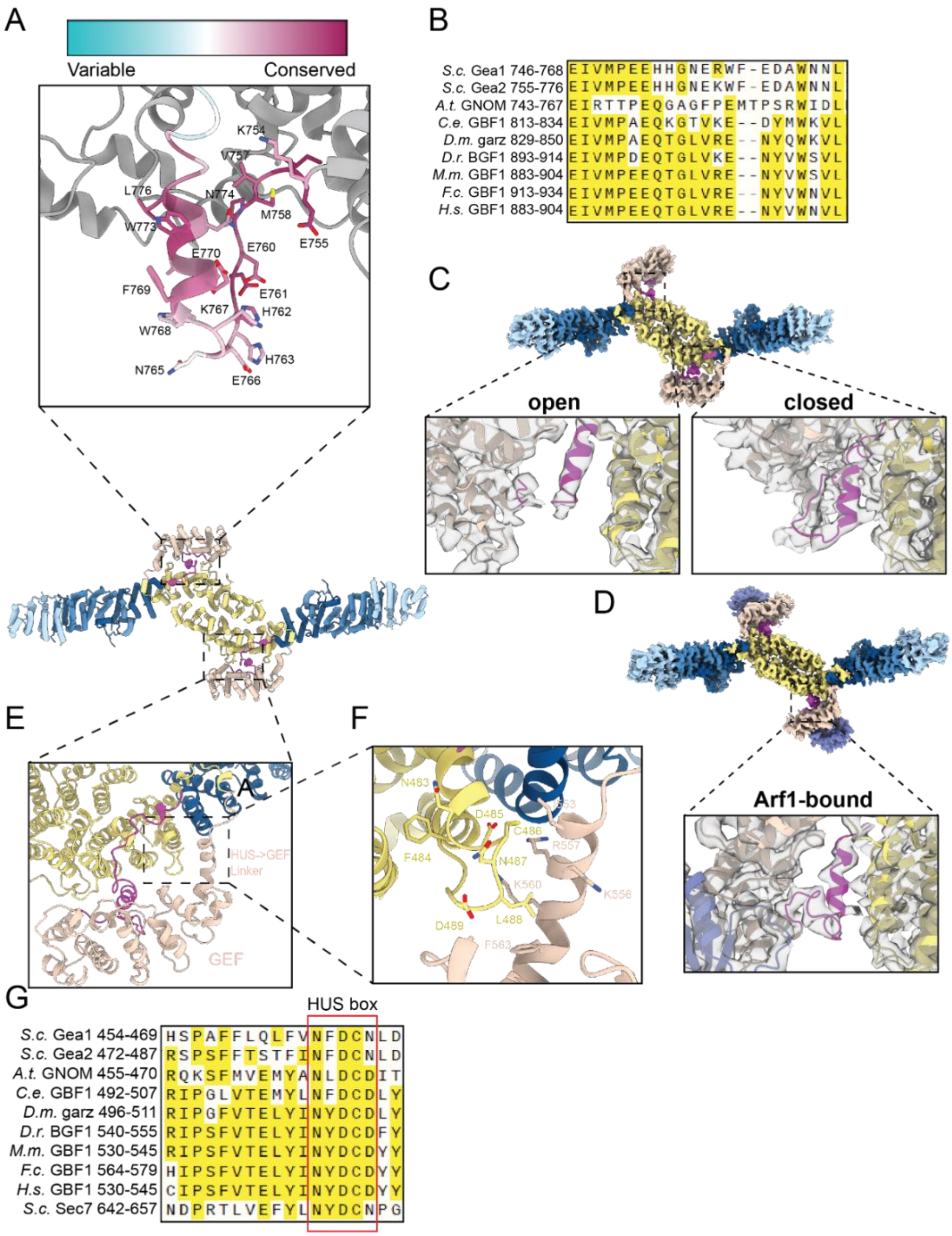

Figure S5. Ordered linkers connect the GEF domain to the HUS and HDS1 domains, Related to Figures 1 and 4

A) Close-up view of the GEF-HDS1 linker in the closed monomer colored by conservation as indicated. B) Sequence alignment of the GEF-HDS1 linker from Gea2 homologs in several model organisms and humans. Yellow highlights indicate identical residues at a given position. C) Views of the GEF-HDS1 linker cryoEM density in the open (left) and closed (right) monomers. D) View of the GEF-HDS1 linker cryoEM density in the Gea2-Arf1 complex. E,F) Close-up views of the HUS-GEF linker and ‘HUS-box’ region in the closed monomer. G) Sequence alignment of the HUS-GEF linker from Gea2 homologs in several models organisms and humans. Yellow highlights indicate identical residues at a given position.

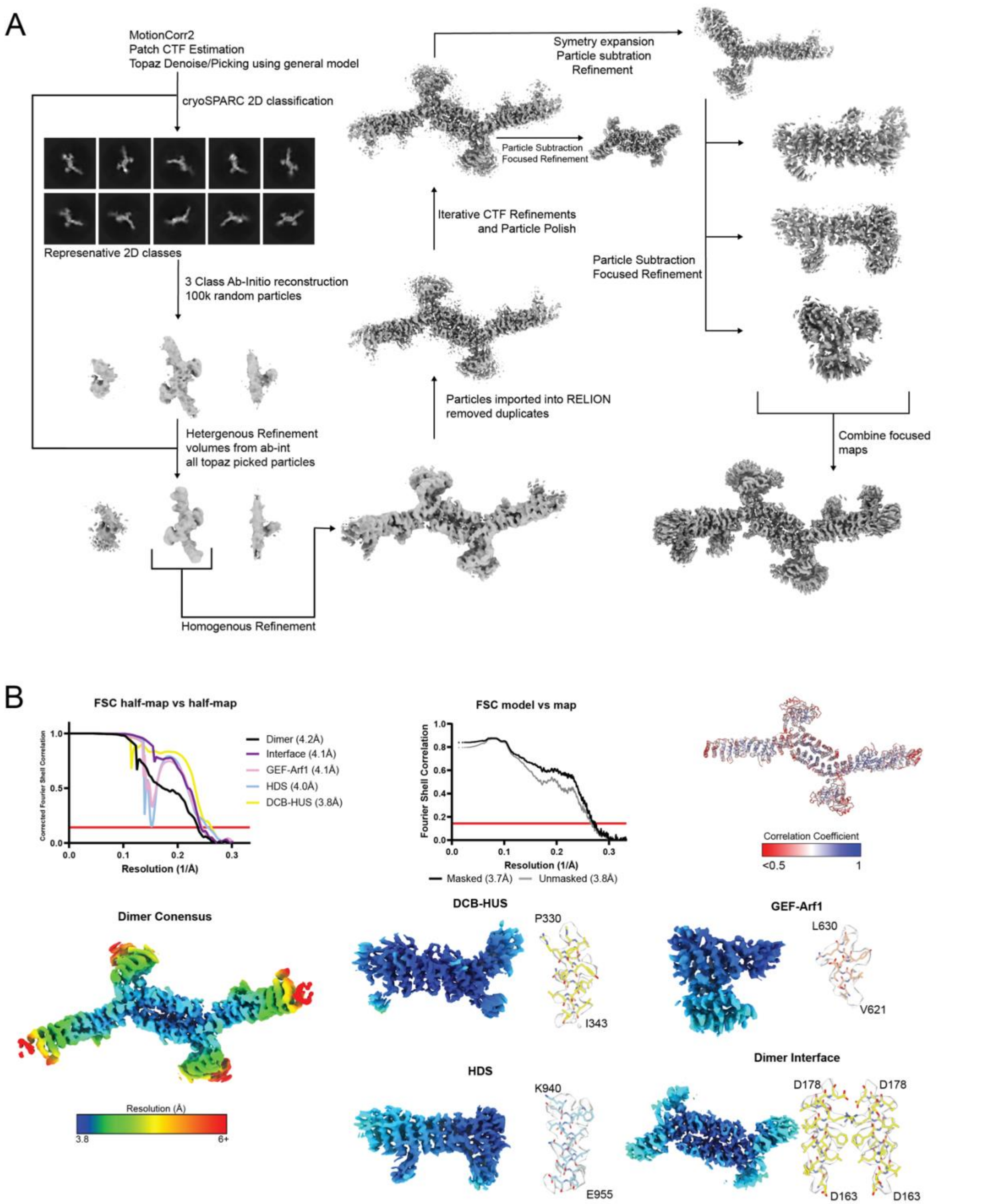

Figure S6. Gea2-Arf1 activation intermediate complex cryoEM data processing, Related to Figure 4 and STAR Methods

A) Flowchart illustrating the data processing workflow for the Gea2-Arf1 complex cryoEM data (see STAR Methods). B) Fourier shell correlation plots and example cryoEM density for focused refinements are shown for the cryoEM map and model of the Gea2-Arf1 complex.

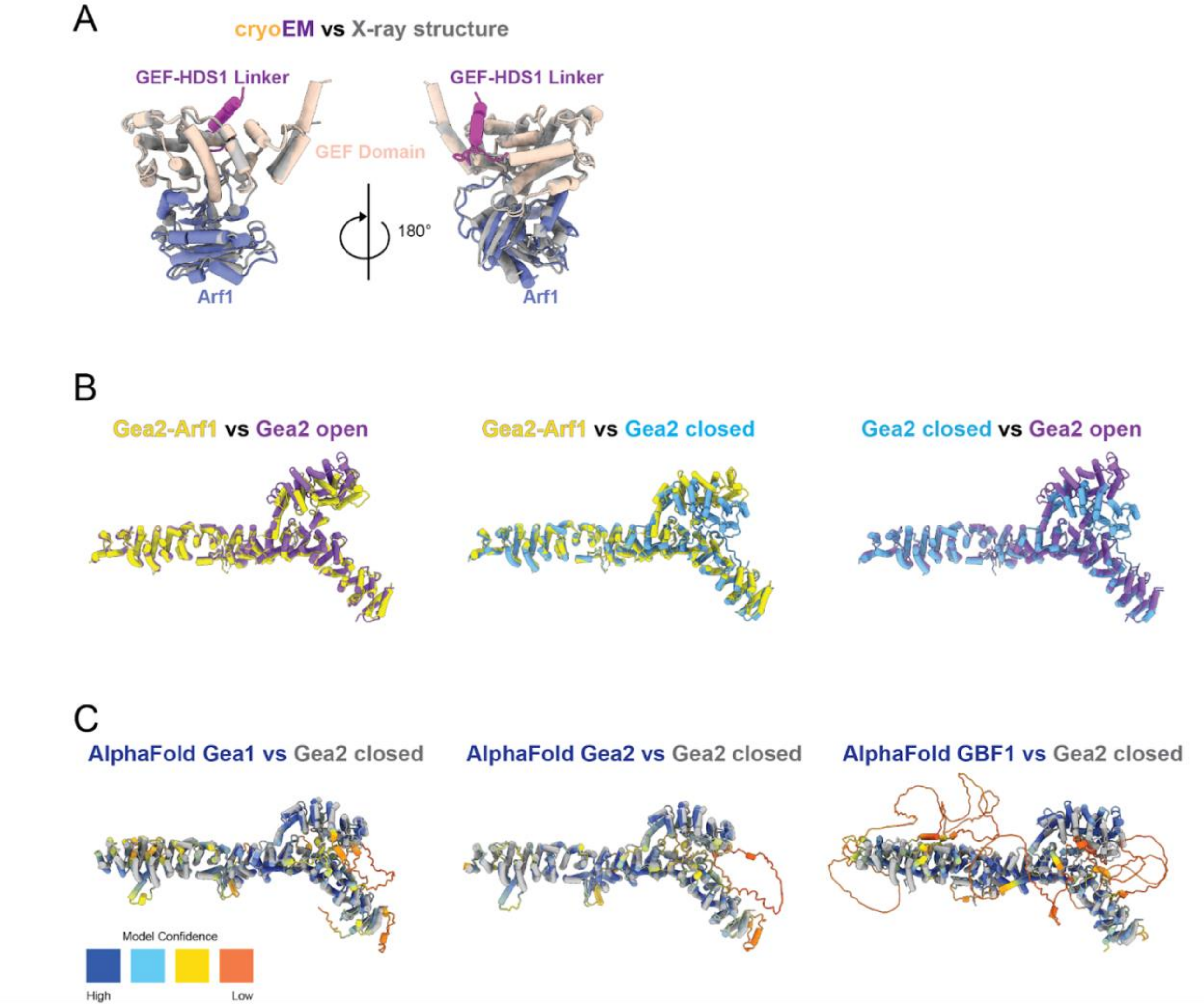

Figure S7. Structural comparisons, Related to Figures 1 and 4

A) CryoEM structure of the Gea2 GEF domain bound to nucleotide-free Arf1 superimposed on the crystal structure of the Gea2 GEF domain bound to nucleotide-free Arf1 (Goldberg, 1998). B) Pairwise superpositions of the Gea2 open, closed, and Arf1-bound monomers. C) Superpositions of the Gea2 closed monomer onto AlphaFold models, colored by prediction confidence, of *S. cerevisiae* Gea1, *S. cerevisiae* Gea2, and human GBF1 (Jumper et al., 2021).
